# 2-Chloro-5-nitro­aniline

**DOI:** 10.1107/S160053680901945X

**Published:** 2009-05-29

**Authors:** Aamer Saeed, Zaman Ashraf, Mahira Batool, Michael Bolte

**Affiliations:** a Department of Chemistry, Quaid-i-Azam University, Islamabad 45320, Pakistan; b Riphah Institute of Pharmaceutical Sciences, Islamabad, Pakistan; c Institut für Anorganische Chemie, J. W. Goethe-Universität Frankfurt, Max-von-Laue-Strasse 7, 60438 Frankfurt/Main, Germany

## Abstract

The mol­ecule of the title compound, C_6_H_5_ClN_2_O_2_, is close to being planar (rms deviation = 0.032 Å for all non-H atoms), with a maximum deviation of −0.107 (3) Å for an O atom. In the crystal structure, inter­molecular N—H⋯O and N—H⋯N inter­actions link the mol­ecules into a three-dimensional network.

## Related literature

For applications of substituted nitro­benzene and aniline derivatives, see: Heinisch *et al.* (1997 ▶); Wang *et al.* (2000 ▶); Yosuke *et al.* (2003 ▶); Zou *et al.* (1997 ▶). For a related structure, see: Zhang *et al.* (2004 ▶). For bond-length data, see: Allen *et al.* (1987 ▶). For synthesis, see: Suwanprasop *et al.* (2003 ▶).
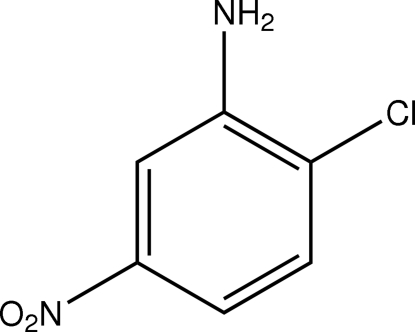


## Experimental

### 

#### Crystal data

C_6_H_5_ClN_2_O_2_
*M* *_r_* = 172.57
Monoclinic, 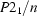

*a* = 13.6233 (10) Å
*b* = 3.7445 (3) Å
*c* = 13.6420 (9) Å
β = 91.768 (5)°
*V* = 695.58 (9) Å^3^
*Z* = 4
Mo *K*α radiation
μ = 0.49 mm^−1^
*T* = 173 K
0.35 × 0.34 × 0.29 mm

#### Data collection

Stoe IPDSII two-circle diffractometer
Absorption correction: multi-scan (*MULABS*; Blessing, 1995 ▶) *T*_min_ = 0.847, *T*_max_ = 0.871
5108 measured reflections
1300 independent reflections
1266 reflections with *I* > 2σ(*I*)
*R*_int_ = 0.034

#### Refinement

*R*[*F* ^2^ > 2σ(*F* ^2^)] = 0.023
*wR*(*F* ^2^) = 0.061
*S* = 1.06
1300 reflections
110 parameters
H atoms treated by a mixture of independent and constrained refinement
Δρ_max_ = 0.20 e Å^−3^
Δρ_min_ = −0.21 e Å^−3^

### 

Data collection: *X-AREA* (Stoe & Cie, 2001 ▶); cell refinement: *X-RED* (Stoe & Cie, 2001 ▶); data reduction: *X-RED*; program(s) used to solve structure: *SHELXS97* (Sheldrick, 2008 ▶); program(s) used to refine structure: *SHELXL97* (Sheldrick, 2008 ▶); molecular graphics: *PLATON* (Spek, 2009 ▶); software used to prepare material for publication: *SHELXL97*.

## Supplementary Material

Crystal structure: contains datablocks global, I. DOI: 10.1107/S160053680901945X/hk2694sup1.cif

Structure factors: contains datablocks I. DOI: 10.1107/S160053680901945X/hk2694Isup2.hkl

Additional supplementary materials:  crystallographic information; 3D view; checkCIF report

## Figures and Tables

**Table 1:** Hydrogen-bond geometry (Å, °)

*D*—H⋯*A*	*D*—H	H⋯*A*	*D*⋯*A*	*D*—H⋯*A*
N2—H2*A*⋯O1^i^	0.85 (2)	2.33 (2)	3.1521 (18)	163.2 (19)
N2—H2*B*⋯N2^ii^	0.88 (3)	2.44 (2)	3.1452 (19)	137.4 (18)

Symmetry codes: (i) 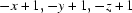; (ii) 
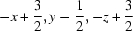
.

**Fig. 1.**
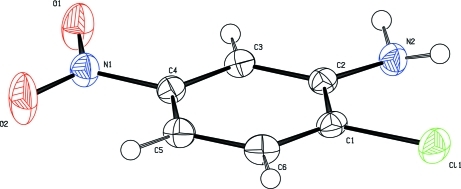
The molecular structure of the title molecule, with the atom-numbering scheme. Displacement ellipsoids are drawn at the 50% probability level.

## supplementary crystallographic information

### Comment

Substituted nitrobenzene and aniline derivatives are valuable as intermediates towards a variety of dye and pigments, heterocycles, pesticides, rubber chemicals and agricultural products, and are useful as textile printing agents, nickel stripping agents and polymerization catalysts. Thus, then title compound is an important intermediate or starting point in the syntheses of alkyl derivatives of 2-aminobenzenethiols substituted by chloro and nitro groups (Wang *et al.*, 2000), donor-bridge-acceptor' triad compounds containing the aromatic sulfur bridges (Yosuke *et al.*, 2003), pyridazinobenzodiazepin-5-ones as non-nucleoside HIV Reverse Transcriptase Inhibitors (Heinisch *et al.*, 1997) and 2-chloro-5,6-dihalo-*D*-ribofuranosylbenzimidazoles as potential agents for human cytomegalovirus infections (Zou *et al.*, 1997). We report herein the crystal structure of the title compound, as a key starting point towards many heterocycles.

In the molecule of the title compound (Fig 1), the bond lengths (Allen *et al.*, 1987) and angles are within normal ranges, and may be compared with the corresponding values in N-tert-butyl-4-chloro-5-methyl-2-nitroaniline (Zhang *et al.*, 2004). Ring A (C1–C6) is, of course, planar. Atoms Cl1, O1, O2, N1 and N2 are 0.019 (3), 0.104 (3), -0.107 (3), 0.003 (3) and -0.066 (3) Å away from the ring plane, respectively. So, the molecule is nearly planar.

In the crystal structure, intermolecular N—H···O and N—H···N hydrogen bonds (Table 1) link the molecules into a network, in which they may be effective in the stabilization of the structure.

### Experimental

The title compound was prepared by nitration and selective reduction of 4-nitroaniline according to the literature method (Suwanprasop *et al.*, 2003). Recrystallization from methanol afforded the title compound. Anal. calcd. for C_6_H_5_Cl_N_2_O_2: C, 41.76; H, 2.92; N, 16.23%; found: C, 41.71; H, 2.97; N, 16.16%

### Refinement

H atoms (for NH_2_) were located in a difference synthesis and refined isotropically. The remaining H atoms were positioned geometrically, with C—H = 0.95 Å for aromatic H and constrained to ride on their parent atoms, with U_iso_(H) = 1.2U_eq_(C).

### Crystal data

**Table:**

C_6_H_5_ClN_2_O_2_	*F*(000) = 352
*M**_r_* = 172.57	*D*_x_ = 1.648 Mg m^−^^3^
Monoclinic, *P*2_1_/*n*	Mo *K*α radiation, λ = 0.71073 Å
Hall symbol: -P 2yn	Cell parameters from 7189 reflections
*a* = 13.6233 (10) Å	θ = 3.4–25.9°
*b* = 3.7445 (3) Å	µ = 0.49 mm^−^^1^
*c* = 13.6420 (9) Å	*T* = 173 K
β = 91.768 (5)°	Block, orange
*V* = 695.58 (9) Å^3^	0.35 × 0.34 × 0.29 mm
*Z* = 4	

### Data collection

**Table:**

Stoe IPDSII two-circle diffractometer	1300 independent reflections
Radiation source: fine-focus sealed tube	1266 reflections with *I* > 2σ(*I*)
graphite	*R*_int_ = 0.034
ω scans	θ_max_ = 25.5°, θ_min_ = 3.4°
Absorption correction: multi-scan (*MULABS*; Blessing, 1995)	*h* = −16→14
*T*_min_ = 0.847, *T*_max_ = 0.871	*k* = −4→4
5108 measured reflections	*l* = −16→16

### Refinement

**Table:**

Refinement on *F*^2^	Secondary atom site location: difference Fourier map
Least-squares matrix: full	Hydrogen site location: inferred from neighbouring sites
*R*[*F*^2^ > 2σ(*F*^2^)] = 0.023	H atoms treated by a mixture of independent and constrained refinement
*wR*(*F*^2^) = 0.061	*w* = 1/[σ^2^(*F*_o_^2^) + (0.0448*P*)^2^ + 0.0466*P*] where *P* = (*F*_o_^2^ + 2*F*_c_^2^)/3
*S* = 1.06	(Δ/σ)_max_ = 0.001
1300 reflections	Δρ_max_ = 0.20 e Å^−^^3^
110 parameters	Δρ_min_ = −0.21 e Å^−^^3^
0 restraints	Extinction correction: *SHELXL97* (Sheldrick, 2008), Fc^*^=kFc[1+0.001xFc^2^λ^3^/sin(2θ)]^-1/4^
Primary atom site location: structure-invariant direct methods	Extinction coefficient: 0.034 (4)

### Special details

**Table:**

Geometry. All e.s.d.'s (except the e.s.d. in the dihedral angle between two l.s. planes) are estimated using the full covariance matrix. The cell e.s.d.'s are taken into account individually in the estimation of e.s.d.'s in distances, angles and torsion angles; correlations between e.s.d.'s in cell parameters are only used when they are defined by crystal symmetry. An approximate (isotropic) treatment of cell e.s.d.'s is used for estimating e.s.d.'s involving l.s. planes.
Refinement. Refinement of *F*^2^ against ALL reflections. The weighted *R*-factor *wR* and goodness of fit *S* are based on *F*^2^, conventional *R*-factors *R* are based on *F*, with *F* set to zero for negative *F*^2^. The threshold expression of *F*^2^ > σ(*F*^2^) is used only for calculating *R*-factors(gt) *etc*. and is not relevant to the choice of reflections for refinement. *R*-factors based on *F*^2^ are statistically about twice as large as those based on *F*, and *R*- factors based on ALL data will be even larger.

### Fractional atomic coordinates and isotropic or equivalent isotropic displacement parameters (Å^2^)

**Table:**

	*x*	*y*	*z*	*U*_iso_*/*U*_eq_	
Cl1	0.91913 (3)	0.59906 (8)	0.62642 (3)	0.02963 (14)	
O1	0.50548 (9)	0.8247 (4)	0.37207 (9)	0.0385 (3)	
O2	0.60383 (10)	1.0787 (4)	0.27322 (9)	0.0475 (4)	
N1	0.58750 (10)	0.9201 (3)	0.34951 (9)	0.0258 (3)	
N2	0.70761 (10)	0.4832 (4)	0.66629 (9)	0.0255 (3)	
H2B	0.7582 (18)	0.367 (5)	0.6920 (18)	0.043 (6)*	
H2A	0.6514 (17)	0.384 (4)	0.6685 (15)	0.029 (5)*	
C1	0.82203 (10)	0.6968 (3)	0.54548 (10)	0.0215 (3)	
C2	0.72634 (11)	0.6173 (3)	0.57428 (10)	0.0203 (3)	
C3	0.64960 (10)	0.6935 (3)	0.50720 (10)	0.0206 (3)	
H3	0.5836	0.6427	0.5230	0.025*	
C4	0.67060 (10)	0.8434 (3)	0.41772 (10)	0.0206 (3)	
C5	0.76477 (11)	0.9244 (3)	0.38893 (11)	0.0231 (3)	
H5	0.7764	1.0285	0.3269	0.028*	
C6	0.84118 (11)	0.8461 (4)	0.45509 (11)	0.0248 (3)	
H6	0.9070	0.8952	0.4383	0.030*	

### Atomic displacement parameters (Å^2^)

**Table:**

	*U*^11^	*U*^22^	*U*^33^	*U*^12^	*U*^13^	*U*^23^
Cl1	0.0223 (2)	0.03330 (19)	0.0329 (2)	0.00210 (13)	−0.00586 (12)	−0.00076 (14)
O1	0.0221 (6)	0.0617 (7)	0.0315 (6)	−0.0054 (5)	−0.0011 (4)	0.0122 (5)
O2	0.0374 (7)	0.0750 (10)	0.0298 (6)	−0.0099 (6)	−0.0040 (5)	0.0271 (6)
N1	0.0252 (7)	0.0302 (6)	0.0218 (6)	−0.0012 (5)	0.0000 (5)	0.0029 (5)
N2	0.0266 (7)	0.0291 (6)	0.0206 (6)	−0.0039 (6)	−0.0011 (5)	0.0041 (5)
C1	0.0215 (7)	0.0184 (5)	0.0245 (7)	0.0006 (5)	−0.0014 (5)	−0.0047 (5)
C2	0.0246 (7)	0.0181 (6)	0.0182 (7)	−0.0004 (5)	0.0014 (5)	−0.0026 (4)
C3	0.0204 (6)	0.0216 (6)	0.0201 (6)	−0.0019 (5)	0.0034 (5)	−0.0021 (5)
C4	0.0222 (7)	0.0196 (6)	0.0198 (6)	0.0001 (5)	0.0006 (5)	−0.0015 (5)
C5	0.0257 (7)	0.0235 (6)	0.0203 (7)	−0.0021 (5)	0.0060 (6)	−0.0007 (5)
C6	0.0199 (7)	0.0247 (6)	0.0301 (7)	−0.0009 (5)	0.0054 (5)	−0.0027 (5)

### Geometric parameters (Å, °)

**Table:**

N1—O1	1.2216 (19)	C2—C3	1.397 (2)
N1—O2	1.2245 (18)	C3—C4	1.382 (2)
N2—H2B	0.88 (3)	C3—H3	0.9500
N2—H2A	0.85 (2)	C4—C5	1.387 (2)
C1—C6	1.386 (2)	C4—N1	1.4713 (18)
C1—C2	1.405 (2)	C5—C6	1.388 (2)
C1—Cl1	1.7357 (14)	C5—H5	0.9500
C2—N2	1.3830 (18)	C6—H6	0.9500
			
O1—N1—O2	123.19 (14)	C4—C3—C2	119.37 (12)
O1—N1—C4	118.40 (12)	C4—C3—H3	120.3
O2—N1—C4	118.41 (13)	C2—C3—H3	120.3
C2—N2—H2B	112.2 (15)	C3—C4—C5	123.90 (13)
C2—N2—H2A	112.4 (14)	C3—C4—N1	117.51 (12)
H2B—N2—H2A	117.7 (18)	C5—C4—N1	118.59 (13)
C6—C1—C2	122.42 (13)	C4—C5—C6	116.84 (13)
C6—C1—Cl1	119.36 (11)	C4—C5—H5	121.6
C2—C1—Cl1	118.22 (11)	C6—C5—H5	121.6
N2—C2—C3	120.88 (13)	C1—C6—C5	120.38 (13)
N2—C2—C1	121.98 (13)	C1—C6—H6	119.8
C3—C2—C1	117.09 (13)	C5—C6—H6	119.8
			
C6—C1—C2—N2	−177.05 (13)	N1—C4—C5—C6	−179.64 (12)
Cl1—C1—C2—N2	3.57 (17)	C2—C1—C6—C5	0.2 (2)
C6—C1—C2—C3	0.33 (19)	Cl1—C1—C6—C5	179.57 (10)
Cl1—C1—C2—C3	−179.04 (9)	C4—C5—C6—C1	−0.5 (2)
N2—C2—C3—C4	176.87 (12)	C3—C4—N1—O1	−5.21 (19)
C1—C2—C3—C4	−0.55 (19)	C5—C4—N1—O1	174.73 (13)
C2—C3—C4—C5	0.2 (2)	C3—C4—N1—O2	174.29 (13)
C2—C3—C4—N1	−179.82 (12)	C5—C4—N1—O2	−5.8 (2)
C3—C4—C5—C6	0.3 (2)		

### Hydrogen-bond geometry (Å, °)

**Table:**

*D*—H···*A*	*D*—H	H···*A*	*D*···*A*	*D*—H···*A*
N2—H2A···O1^i^	0.85 (2)	2.33 (2)	3.1521 (18)	163.2 (19)
N2—H2B···N2^ii^	0.88 (3)	2.44 (2)	3.1452 (19)	137.4 (18)

Symmetry codes: (i) −*x*+1, −*y*+1, −*z*+1; (ii) −*x*+3/2, *y*−1/2, −*z*+3/2.
